# Attitudes towards help-seeking for sexual and gender-based violence in humanitarian settings: the case of Rwamwanja refugee settlement scheme in Uganda

**DOI:** 10.1186/s12914-018-0154-6

**Published:** 2018-03-12

**Authors:** George Odwe, Chi-Chi Undie, Francis Obare

**Affiliations:** Population Council, P. O. Box 17643 -00500, Nairobi, Kenya

**Keywords:** Sexual and gender-based violence, Help-seeking, Attitudes and behavior, Humanitarian settings, Uganda

## Abstract

**Background:**

Sexual and gender-based violence (SGBV) remains a silent epidemic in many humanitarian settings with many survivors concealing their experiences. Attitudes towards help-seeking for SGBV is an important determinant of SGBV service use. This paper examined the association between attitudes towards seeking care and knowledge and perceptions about SGBV among men and women in a humanitarian setting in Uganda.

**Methods:**

A cross-sectional survey was conducted from May to June 2015 among 601 heads of refugee households (261 females and 340 males) in Rwamwanja Refugees Settlement Scheme, South West Uganda. Analysis entails cross-tabulation with chi-square test and estimation of a multivariate logistic regression model.

**Results:**

Results showed increased odds of having a favorable attitude toward seeking help for SGBV among women with progressive attitudes towards SGBV (OR = 2.78, 95% CI: 1.56–4.95); who felt that SBGV was not tolerated in the community (OR = 2.03, 95% CI: 1.03–4.00); those who had not experienced violence (OR = 2.08, 95% CI: 1.06–4.07); and those who were aware of the timing for post-exposure prophylaxis (OR = 3.08, 95% CI: 1.57–6.04). In contrast, results for men sample showed lack of variations in attitude toward seeking help for SGBV for all independent variables except timing for PEP (OR = 2.57, 95% CI: 1.30–5.10). Among individuals who had experienced SGBV, the odds of seeking help was more likely among those with favorable attitude towards seeking help (OR = 4.22, 95% CI: 1.47–12.06) than among those with unfavorable help-seeking attitudes.

**Conclusion:**

The findings of the paper suggest that targeted interventions aimed at promoting awareness and progressive attitudes towards SGBV are likely to encourage positive help-seeking attitudes and behaviors in humanitarian contexts.

## Background

Globally, approximately one-third of women have experienced Sexual and Gender-Based Violence (SGBV)—defined as any harm imposed on a person on the basis of gender and unequal power relationships [1]. SGBV has also been recognized as a growing problem in humanitarian settings [2–5]. It is associated with a wide range of physical, sexual and psychological health consequences [6, 7]. Studies have also shown negative impacts of SGBV on the social and economic well-being of survivors [8, 9]. These outcomes are particularly exacerbated in humanitarian settings given that crisis-affected populations are more vulnerable to SGBV [10].

Approximately 1 in 5 female refugees have experienced sexual violence [11]. However, the number could be higher given that many survivors are hesitant to disclose. Despite SGBV being pervasive among conflict-affected populations and within humanitarian settings, the rate of SGBV disclosure and seeking services is quite low [12, 13]. Studies show that attitude towards seeking help for SGBV is an important predictor of utilization of formal and informal services among survivors [14, 15]. However, there is limited evidence on factors associated with help-seeking attitudes for SGBV in humanitarian settings. The paucity in documented evidence is attributed to lack of reliable data [16], and barriers associated with SGBV disclosure such as stigma, shame, fear of reprisal, lack of services and inadequate capacity of service providers to respond to the needs of survivors [11, 17, 18].

Various social and cultural barriers may influence attitude towards seeking help for SGBV, and further contribute to underutilization of SGBV services in emergency settings [15, 19]. Research has shown that attitudes and perceptions towards SGBV norms influence disclosure and help-seeking [20]. Individuals with violent-supportive attitudes are more likely to hold negative help-seeking attitude. Help-seeking for SGBV is also notably low in communities where sexual violence is tolerated or considered as a normal act [21]. In addition to attitudinal barriers, research shows that personal exposure to SGBV and its severity may also influence attitudes toward reporting or seeking help [20, 22, 23]. Past survivors tend to have a favorable attitude towards help-seeking for SGBV; however, this may be influenced by their past experience with services. Other important factors that may influence help-seeking for SGBV include perceived benefits of seeking help and distrust of service providers [24].

Individual attributes such as sex, age, marital status, and education have also been shown to be important predictors of attitudes towards seeking help for SGBV [25, 26]. Research shows that men, compared to women, are less likely to report or seek help from formal sources such as the police, health professionals or community resource centers [23]. A positive correlation exist between age and a favorable attitude towards seeking help for SGBV—mainly due to increased autonomy [16]. Education has also been shown to be positively associated with a favorable attitude towards seeking help for SGBV. The impact of education is mainly through increased awareness, autonomy and economic empowerment among those with high levels of education [25–27].

**Fig. 1:**
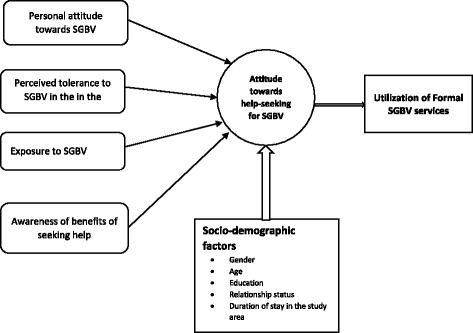
Conceptual framework

This paper examines factors associated with attitude towards seeking formal help for SGBV from health facilities and law-enforcement agencies, and test whether such attitudes are associated with actual help-seeking in a humanitarian setting in Uganda. The hypothesis is that a positive attitude toward help-seeking for SGBV will more likely be found among individuals with progressive SGBV norms (i.e. do not perceive violence as a normal act), those who do not perceive SGBV to be tolerated in their community, those who have experienced it before, and those who are aware of the benefits of seeking help. Figure 1 presents a conceptual framework for understanding factors associated with attitude towards seeking help for SGBV [28]. Attitudes towards help-seeking for SGBV in this context refers to the tendency to be supportive (or unsupportive) of disclosure or of the reporting of SGBV cases in order to receive appropriate services [21, 29]. Exploring factors associated with attitudes towards seeking help for SGBV is important for improving policies and programmes for prevention and response.

## Methods

### Study design

The data for this paper come from a cross-sectional baseline survey that was aimed at generating evidence to inform a community-based intervention to prevent SGBV in an emergency context in Uganda. A detailed study and intervention design may be found elsewhere [30].

### Setting

The study was conducted in Rwamwanja Refugee Settlement scheme in Kamwenge District, South West Uganda. The settlement scheme was established in 1963–64 to cater for populations fleeing civil strife in neighboring countries. The Ugandan policy for refugees upholds key rights, including freedom of movement and expression, and favors a settlement approach as opposed to a camp model. Under this policy, registered refugees are provided with land—usually 1.25 acres per household, for housing and farming. Thus, settled refugees have means of self-sustainability, and engage in local trade with host communities.

Rwamwanja settlement scheme covers an area of about 108.5 km^2^ divided into 15 zones and 45 villages. By the end of 2015, the population of the scheme was estimated to be 40,000 people. However, the population was expected to increase to a maximum capacity of between 55,000 and 60,000 due to escalating violence in neighboring countries. SGBV is a challenge in the settlement area. In a community baseline survey carried out in the study site in 2015, a large proportion of female household heads reported experiencing physical IPV (69%) and sexual IPV (63%) since moving into the settlement area—the majority (68%) had lived in the area for more than 2 years [30]. Furthermore, many female heads of household indicated that they had experienced non-partner physical violence (47%) and non-partner sexual violence (46%) since they started residing in the settlement. Data from two health facilities in the scheme showed that 837 (10%) out of 8462 female clients (aged 15 and above) screened by health providers from September 2015 to January 2016, had been exposed to some form of SGBV including intimate partner violence (IPV) and/or non-partner sexual violence [31].

### Participants

Participants were male and female heads of refugee households aged between 12 and 49 years residing in Rwamwanja settlement scheme. The inclusion of only household heads was a deliberate decision due to: (1) ethical concerns related to the sensitivity of SGBV, (2) the high prevalence of SGBV in the study area; and (3) the need to ensure protection of female interviewees [32]—female household heads were presumed to have greater autonomy over their own decisions (e.g., decision to participate in an interview), and were therefore seen as less likely to suffer SGBV as a result of participating in the study compared with female refugees whose households were headed by men.

### Sampling

Participants were identified in stages. In the first stage, one zone (Kyempango A) was purposely selected in collaboration with the United Nations High Commissioner for Refugees (UNHCR), based on high rates of reported SGBV cases in the area [33]. Two villages within this zone (Village A1 and Village A3) were then randomly selected out of five villages in the second stage. The third stage involved identification of heads of household for interview. All households in each of the sampled villages were targeted, and every household head available was invited to participate in an interview. The sampling criteria was informed by the noted high movement of population in and out of the study area as well as in the overall Settlement scheme. In each household, only one female or male member of refugee status, and self-reporting as the head of a household was eligible for interview. Heads of household aged less than 18 were considered emancipated minors. A total of 601 household heads (340 males and 261 females) were interviewed. Heads of household aged less than 15 years were excluded from analysis.

### Data collection

Data collection took place from May to June 2015 using a structured questionnaire. A considerable proportion of the questions were adapted from the GEM (Gender Equitable Men) Scale [34] and WHO multi-country study on women’s health and domestic violence against women [35] The questionnaire captured information on basic socio-demographic characteristics (gender, age, educational attainment, and employment status), access to information and support services for SGBV, experience and perpetration of SGBV, knowledge and use of SGBV services, and attitudes toward seeking help for SGBV. The questionnaire was prepared in both English and Kiswahili languages. The questions posed to male and female respondents were similar, except for a few that were irrelevant for men (e.g. questions on pregnancy experience).

The survey was administered by a team of five research assistants (2 males and 3 females) with experience in collecting sexual and reproductive health-related data and had worked in refugee settings before. Respondents were interviewed by same-sex interviewer. The survey tool was pre-tested and adjusted to suit the study context. In addition, the research assistants were trained on the goals of the project, data collection, and ethics over a one-week period.

The questionnaire was programmed in Open Data Kit (ODK)—an open source data management software—while data capture was conducted using tablets. Each interview lasted 30 min on average and was checked for completeness at the end of each day during data collection period. Data were stored in a computer with restricted access and also sent to a central database server at managed by the Population Council, Nairobi office. The data were then exported into Stata for cleaning and analysis.

### Ethics

The WHO ethical and safety recommendations for researching, documenting and monitoring sexual violence in emergencies were followed [5]. Individual written informed consent was obtained from all participants before conducting the interviews. Interviews were conducted in private and in a non-judgmental manner. Participants were also referred for SGBV services, when necessary.

### Measurements

The analysis focused on assessing the relationship between attitude towards seeking help for SGBV on the one hand, and knowledge, perceptions and experiences of SGBV in the community on the other, controlling for respondents’ socio-demographic characteristics. Table 1 presents measurement of attitude towards seeking help for SGBV, attitude towards SGBV, perceived tolerance of SGBV in the community, and exposure to violence variables.

**Table 1:** Measurement of Attitude towards seeking help for SGBV, Attitude towards SGBV, Perceived tolerance of SGBV in the community and Exposure to Violence

	Source	Cronbach’s Alpha
Attitude towards seeking help for SGBV		
1. When one has been forced to have sex against their will, it is important to report this to the health center immediately. 2. When one has been forced to have sex against their will, it is worth it to report this to the health center immediately. 3. When one has been forced to have sex against their will, it is important to report this to the police immediately. 4. When one has been forced to have sex against their will, it is worth it to report this to the police immediately.	Developed by authors based on UNCHR guidelines for prevention and response to SGBV in emergency settings [36]	0.64
Attitude towards SGBV		
1. Men are justified in hitting or beating their wives/partners/girlfriends when they are angry. 2. Men are justified in hitting or beating their wives/partners/girlfriends when they are denied sex. 3. Men are justified in hitting or beating their wives/partners/girlfriends when they are denied food. 4. Men are justified in hitting or beating their wives/partners/girlfriends when they are denied money.	Adapted from GEM scale items on violence [34]	0.66
Perceived tolerance of SGBV in the community		
In Kyempango A settlement… 1. the community does not tolerate rape. 2. the community does not tolerate wife beating. 3. the community does not tolerate the beating up of others. 4. it is normal for women and/or girls to be forced to have sex. 5. it is normal for women and/or girls to get beaten up. 6. it is normal for men and/or boys to be forced to have sex. 7. it is normal for men and/or boys to get beaten up.	Authors	0.68
Experiences of partner or non-partner violence		
1. Does your partner ever hurt you physically in any way (e.g., beat you up, slap/punch/kick/push/drag you)? 2. Does your partner ever force you to have sex with him even when you do not want to? 3. Since you started living in the study area, has anyone [other than your partner] ever hurt you physically in any way (e.g., beat you up, slapped /punched/kicked/pushed/dragged you)? 4. Since you started living in the study area, has anyone [other than your partner] ever forced you to have sex against your will?	Adapted from WHO Multi-country Study on Women’s Health and Domestic Violence against Women [35]	

#### Attitude towards help-seeking for SGBV

Attitude towards help-seeking for SGBV was the outcome variable. Due to lack of standardized instrument assessing attitude towards help-seeking for SGBV among refugees’ populations, we conceptualized the outcome variable based on the UNCHR guidelines for prevention and response to SGBV in emergency settings [36]. The guideline underscores the need for survivors to report incidents of sexual and gender-based violence so as to receive adequate care and support. Attitude towards help-seeking for SGBV was measured based on a series of 4 statements that asked participants to indicate their perceptions of the importance, and value of seeking help for SGBV at health facilities, and police stations (Table 1). The response categories were *1 = agree, 2 = disagree and 3 = not sure*. Factor analysis was performed to generate scores for attitude towards seeking help for SGBV. The measure was found to be reliable (Cronbach’s alpha = 0.64). A binary outcome variable—attitude towards help-seeking for SGBV—was created from the scores to represent those with favorable attitude (that is, respondents indicating that it is important and worth it for someone who has experienced rape to report to a health center or police immediately), or otherwise. The analytical approach was similar to that used in other studies to measure attitude towards help-seeking for SGBV [26, 37]. Respondents with scores above zero were categorized as having a favorable attitude towards help-seeking for SGBV while those scoring zero and below were considered to have unfavorable help-seeking attitudes.

#### Attitude towards SGBV

Attitude towards SGBV was measured through a composite variable summing responses to 4 items on views regarding hypothetical gender norms in intimate partnerships adapted from GEM scale items on violence [34]. Respondents were asked to indicate whether they agreed, disagreed, or were not sure about the justifiability of men’s physical violence towards their partners if it resulted from their being angry, denied sex, food, or money (Table 1). Factor analysis was performed to generate scores for attitude towards SGBV. The items were coded such that higher score represented a progressive attitude (disagreement with all four statements on acceptability of IPV) while the lower score represented regressive attitudes (agreement with all four statements on acceptability of IPV). The measure had an internal reliability coefficient (Cronbach alpha = 0.66). A binary variable was then generated from the score where respondents who scored zero and below were considered to have a regressive attitude towards SGBV while those who scored above zero had a progressive attitude towards SGBV.

#### Perceived tolerance of SGBV in the community

This was assessed based on a series of 7 statements centering on respondents’ perceptions of tolerance violence in the study area. Specifically, respondents were asked to indicate whether they agreed, disagreed, or were not sure if rape, wife-beating and physical violence toward others were tolerated or considered normal in their community (Table 1). Factor analysis was performed to generate scores for perceived tolerance of SGBV in the community (Cronbach’s alpha = 0.68). A binary variable was then generated from the scores such that respondents scoring zero and below were considered to view SGBV as not being tolerated in the community while those with scores above zero felt otherwise.

#### Experiences of partner or non-partner violence

It was measured based on 4 questions that asked whether a respondent had ever experienced physical or sexual violence- whether it originated from a partner or non-partner (Table 1). For non-partner violence (both sexual and physical), respondents were asked if it had happened since they started residing in the study area. Due to repetitive nature of IPV, respondents were asked if their partners ever hurt them physically or force them to have sex against their wish. A binary variable was created to capture exposure to partner or non-partner violence i.e. whether the respondent had ever experienced physical or sexual violence perpetrated by an intimate partner and/or non-intimate partner (since they started residing in the study site), or otherwise.

#### Knowledge of timing for post-exposure prophylaxis (PEP)

Survivors of sexual abuse can suffer a wide range of health problems such as sexually transmitted diseases (STDs), HIV/AIDS as well as unwanted pregnancy. The World Health Organization (WHO) recommends provision of preventive anti-retroviral therapy or HIV post-exposure prophylaxis to sexual violence survivors within 72 h of exposure to STIs and HIV [38]. However, lack of awareness about the 72-h window for post-exposure prophylaxis may reduce the likelihood that survivors seek immediate medical care. Knowledge of the timing for PEP was used to measure awareness of the benefits of seeking help. It was assessed by asking respondents whether they thought it was true or false for one who has experienced coercive sex to report this to a health center or the police within 72 h of the incident. Knowledge of timing for PEP was coded as 1 ‘aware’ and 0 ‘otherwise’.

#### Sociodemographic characteristics

Analysis controlled for respondents’ age (in grouped years as 1 = ‘15–24′ 2 = ‘25–34′ 3 = ‘35–44′ 4 = ‘45 +’), sex (coded as 1 = male and 2 = female), educational level (coded 1 = no education, 2 = primary school education and 3 = secondary school education and above), and whether they were in an intimate relationship.

### Analysis

Analysis entailed a cross-tabulation with Chi-square test to examine statistically significant relationship between attitude towards help-seeking for SGBV and independent variables. A multivariate logistic regression model was fitted to examine factors associated with attitude towards and actual help-seeking for SGBV. The results are presented as Odds Ratios (OR) with 95% confidence intervals (CI). A *p*-value of < 0.05 was considered statistically significant. Data analysis was performed using Stata statistical software version 11. Cronbach’s alpha measures were computed to check for internal consistency for all composite variables [39].

## Results

### Characteristics of respondents

The proportion of men was higher than women, which reflects the fact that household headship has conventionally been a male preserve in such settings (Table 2). The majority of men and women were aged 25–34 years, had lived in the study area for more than 2 years, perceived that SGBV was not tolerated in their community and were aware of the timing for PEP. The proportion of women with no formal education was twice that of men (69% and 34%, respectively). In terms of exposure to violence, more women (72%) than men (32%) had ever experienced partner or non-partner (in the study area). Furthermore, women were more likely than men to express regressive attitudes towards SGBV and unfavorable attitude towards help-seeking for SGBV.

**Table 2:** Distribution of study participants by background characteristics

	Heads of household		
	Men	Women	All
	(*N* = 340)	(*N* = 259)	(*N* = 599)
Background characteristics	%	%	%
Age group			
15–24	16.9	29.7	22.2
25–34	36.4	38.2	37.2
35–44	26.0	20.9	23.9
45+	20.7	11.2	16.7
Level of education			
No education	33.9	68.7	48.9
Primary	47.2	26.6	38.4
Secondary+	18.9	4.6	12.7
In an intimate relationship			
Yes	72.6	13.5	47.1
No	27.4	86.5	52.9
Duration of stay			
Less than 2 years	30.1	35.1	32.2
2 years or more	69.9	64.9	67.8
Attitude towards SGBV			
Progressive	79.7	43.6	63.9
Regressive	20.4	56.4	36.1
Perceived tolerance of SGBV in the community			
Tolerated	22.4	24.3	23.4
Not tolerated	77.6	75.7	76.6
Ever experienced partner or non-partner violence			
Yes	31.9	72.2	49.2
No	68.1	27.8	50.8
Awareness of the timing for PEP			
Not aware	73.2	76.8	74.8
Aware	26.8	23.2	25.2
Attitude towards help-seeking for SGBV			
Favorable	86.4	52.5	71.7
Unfavorable	13.6	47.5	28.3

### Bivariate analysis

Table 3 presents a cross-tabulation between attitude towards help-seeking for SGBV and selected background variables for men and women. Overall, duration of stay in the study area, attitude towards SGBV, exposure to violence and the perception of tolerance of SGBV in the community were found to be significantly associated with attitude towards seeking help for SGBV among women but not among men. Women who had live in the study area for less than 2 years, had expressed progressive attitude towards SGBV, perceived that SGBV was not tolerated in the community and had not experienced violence were more likely to express favorable help-seeking attitude for SGBV. Awareness of the timing for PEP was significantly associated favorable help-seeking attitude for SGBV for both men and women (*p* < 0.001).

**Table 3:** Proportion of respondents with favorable attitude towards help-seeking for SGBV by background characteristics

	Head of households					
	Men			Women		
	%	*N*	*p*-value	%	*N*	*p*-value
Age group						
15–24	79.0	56		54.6	77	
25–34	86.2	124	0.231	52.5	99	0.965
35–44	87.5	89		50.0	54	
45+	91.4	71		51.7	29	
Level of education						
No education	80.9	115		50.0	178	
Primary	88.1	161	0.073	56.5	69	0.395
Secondary+	92.2	64		66.7	12	
In an intimate relationship						
Yes	85.4	247		48.6	35	
No	89.3	93	0.352	53.1	224	0.616
Duration of stay						
Less than 2 years	85.3	103		64.8	91	
2 years or more	86.9	237	0.689	45.8	168	0.003
Attitude towards SGBV						
Progressive	86.7	270		68.1	113	
Regressive	85.5	70	0.802	40.4	146	0.000
Perceived tolerance of SGBV in the community						
Tolerated	84.2	77		36.5	63	
Not tolerated	87.1	263	0.251	57.7	196	0.003
Ever experienced partner or non-partner violence						
Yes	89.8	108		46.5	187	
No	84.9	232	0.213	68.1	72	0.002
Awareness of timing for PEP						
Not aware	89.9	248		58.8	199	
Aware	76.9	92	0.002	31.7	60	0.000

### Factors associated with favorable attitude towards help-seeking for SGBV

Table 4 presents result from a multivariate logistic regression model showing factors associated with attitude towards seeking help for SGBV for men and women. Women who held progressive attitude towards SGBV were significantly more likely to report a favorable attitude towards help-seeking for SGBV (OR 2.78, CI = 1.56–4.95) compared to those with regressive attitude. The results further show that women who perceived that SGBV was not tolerated in the community were about 2.03 times (CI = 1.03–4.00) more likely to indicate a favorable help-seeking attitude for SGBV compared to women who believed that SGBV was tolerated in their community (Table 4 and 5).

**Table 4:** Adjusted Odds ratios from a multivariate logistic regression model showing factors associated with favorable attitudes towards help-seeking for SGBV among men and women

	Men			Women		
Predictor	OR	[95% CI]	*P* > z	OR	[95% CI]	*P* > z
Age group						
15–24^a^	1.00			1.00		
25–34	1.96 [0.80–4.79]		0.140	0.64 [0.32–1.28]		0.206
35–44	2.09 [0.80–5.47]		0.134	0.76 [0.34–1.66]		0.486
45+	3.45 [1.14–10.4]		0.028	0.31 [0.11–0.88]		0.027
Level of education						
No education^a^	1.00			1.00		
Primary	1.70 [0.84–3.47]		0.143	1.03 [0.55–1.92]		0.922
Secondary+	2.20 [0.74–6.51]		0.155	1.28 [0.29–5.64]		0.747
In intimate relationship						
No^a^	1.00			1.00		
Yes	1.49 [0.66–3.38]		0.337	0.84 [0.37–1.90]		0.672
Duration of stay						
<=2 years^a^	1.00			1.00		
> 2 years	1.11 [0.54–2.28]		0.780	0.66 [0.37–1.18]		0.163
Attitudes towards SGBV						
Progressive^a^	1.00			1.00		
Regressive	1.15 [0.51–2.60]		0.730	2.78 [1.56–4.95]		0.001
Perceived tolerance of SGBV in the community						
Tolerated^a^	1.00			1.00		
Not tolerated	1.28 [0.59–2.77]		0.528	2.03 [1.03–4.00]		0.042
Experiences of partner or non-partner violence						
Yes^a^	1.00			1.00		
No	0.66 [0.31–1.42]		0.289	2.08 [1.06–4.07]		0.034
Knows timing for PEP						
No^a^	1.00			1.00		
Yes	2.57 [1.30–5.10]		0.007	3.08 [1.57–6.04]		0.001

Note: ^a^reference category

**Table 5:** Adjusted Odds ratios from a multivariate logistic regression model showing factors associated with help-seeking behavior for SGBV among men and women (*N* = 109)

Predictor	OR	95%CI		*P* > z
Attitude towards help-seeking for SGBV				
Unfavorable^a^	1.00			
Favorable	4.22	1.47–12.06		0.007
Sex				
Men^a^	1.00			
Women	0.78	0.30–2.07		0.622
Duration of stay				
Less than 2 years^a^	1.00			
2 years or more	1.35	0.54–3.39		0.519
Attitudes towards SGBV				
Regressive^a^	1.00			
Progressive	2.90	1.02–8.49		0.046
Perceived tolerance of SGBV in the community				
Tolerated^a^	1.00			
Not tolerated	4.00	1.24–12.9		0.021
Awareness of timing for PEP				
Not aware^a^	1.00			
Aware	1.80	0.61–5.36		0.288

Note: ^a^reference category

Contrary to expectations, women who had not experienced violence were significantly more likely (OR = 2.08, CI = 1.06–4.07) to express a favorable attitude towards help-seeking for SGBV compared to those who had had previous experience of partner or non-partner violence. The results further showed that the odds of reporting a favorable attitude towards help-seeking for SGBV was 3.08 (CI = 1.57–6.04) times higher among women who were aware of the appropriate timing for PEP in case of coercive sex compared to those unaware.

Similar to women, men who were aware of the appropriate timing for PEP in case of coercive sex were significantly more likely (OR = 2.57, CI = 1.30–5.10) to express a favorable attitude towards help-seeking for SGBV compared to those unaware. The result, however, did not show any statistically significant variation in attitude towards help-seeking for SGBV by attitudinal/perception variables (attitude towards SGBV and perceived tolerance of SGBV in the community) and experience partner and non-partner violence among men (Table 4).

The result showed that there was no statistically significant association between attitude towards help-seeking for SGBV and socio-demographic attributes—age, level of education, being in an intimate relationship with SGBV, and duration of stay in the study area for men and women (Table 4). However, the relationship between the level of education and attitude towards help-seeking for SGBV was in the expected direction—that is, respondents with primary and secondary level of education were more likely than those with no education to express a favorable attitude towards help-seeking for SGBV.

### Attitude towards and actual help-seeking for SGBV

**Fig. 2:**
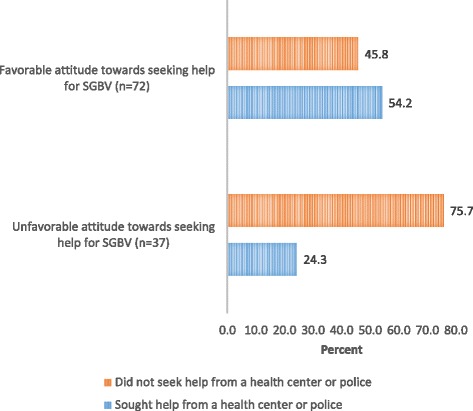
Association between attitudes towards and actual help-seeking for SGBV

The final part of analysis examined the association between attitude towards and actual help-seeking behavior among respondents exposed to SGBV in the study area. All respondents who reported experiencing sexual or physical violence in the last one month preceding the interview—whether it originated from partner or non-partner—were further asked to indicate whether they reported the incident to the police or sought medical care. A total of 109 (18.2%) out of 599 respondents reported experiencing either partner or non-partner violence. The proportion of survivors seeking formal help was significantly higher among those with a favorable attitude towards help-seeking for SGBV compared to those without (54% and 24% respectively; *p* < 0.005; Fig. 2).

To test for the statistical significance of the association between attitudes towards and actual help-seeking for SGBV, a second logistic regression model was fitted with the actual help-seeking behavior as the outcome variable. The low sample size limited a separate analysis for men and women and the inclusion of all variables. Only variables that had a statistically significant association with attitude towards help-seeking for SGBV (as shown in Table 3) were included in the final model. The odds of seeking help for SGBV increased as much as 4 times for survivors with a favorable attitude towards help-seeking for SGBV compared to those with unfavorable attitude. Survivors with a progressive attitude towards SGBV attitude were significantly more likely to seek help for SGBV compares to those with regressive attitude towards SGBV (OR = 2.9; CI = 1.02–12.9). The results further show that survivors who perceived that SGBV was not tolerated in the community were 4 times more likely to seek help for SGBV compared to those who believed that SGBV was a normal occurrence or tolerated in the community (OR =4.00; CI = 1.24–12.9).

## Discussion

This paper examined factors associated with attitudes towards seeking help for SGBV in a humanitarian setting in Uganda. It specifically focused on perceptions of SGBV norms, knowledge, and experiences of SGBV as they relate to attitudes towards seeking help for SGBV, and whether such attitudes translate into actual help-seeking or otherwise.

One major finding of the paper is that views regarding SGBV in humanitarian settings are important for shaping the attitudes towards seeking help from formal sources (medical and police). In particular, women who felt that SGBV is unjustifiable are more likely than those with contrary views to express a favorable attitude towards seeking help for SGBV. However, the fact that a substantial proportion women (56%) compared to men (20%) held regressive attitudes towards SGBV (i.e. had indicated that men’s physical violence toward their partners was justified if it resulted from their being angry – or from their being denied sex, food, or money) suggests the need for targeted interventions aimed at creating awareness around the potential negative impact of such views in an already volatile setting.

As expected, women who felt that SGBV was not tolerated in the community were significantly more likely to express a favorable attitude towards seeking help for SGBV compared to those who felt otherwise. Previous studies show that perception of violence as a ‘normal act’ has a negative influence on attitudes towards help-seeking, which in turn directly or indirectly hinder utilization of SGBV services [21, 26]. The prevailing attitudes and beliefs around SGBV have important implications for how individuals respond to the practice either as victims or witnesses. In addition, the belief that men’s physical violence toward their partners is justified may prevent some of the survivors from seeking help. Individuals who perceive that SGBV is tolerated in the community may not seek help under the belief that such actions may not change the behaviors of the perpetrators or community attitudes and beliefs regarding the practice. The findings suggest the need for context-specific information, education and communications (IEC) interventions aimed at addressing retrogressive norms in an already volatile environment such as the refugee settlement scheme in Uganda.

Previous studies show that personal exposure to SGBV encourages reporting and help-seeking for SGBV [15, 23]; hence, it would be expected that individuals who have been exposed to SGBV would be more likely than those who are non-exposed to have favorable attitudes towards help-seeking. However, result shows that exposure to SGBV was negatively associated with a favorable attitude towards seeking help in the study area. The finding may partly be due to the rationalization of prior behaviors—that is, respondents may have tried to justify their failure to seek care in the past [40]. To the extent that this was the case, the finding would be consistent with the cognitive dissonance framework which posits that people can alter their attitudes to justify their past behavior [41]. The unexpected relationship may also be attributed to survivors’ previous experience with help-seeking. Research shows that survivors of SGBV may develop a negative attitude towards disclosure as a result of a previous bad experience with help-seeking [24].

Another finding of the paper is that knowledge about the timing for PEP was significantly associated with a favorable attitude toward help-seeking for SGBV for both men and women. Research shows that sexual violence is associated with negative health outcomes including; unwanted pregnancy, physical trauma, mental distress, HIV/AIDS and other sexually-transmitted infections [1, 7]. As a result, PEP is recommended within 72 h following unwanted sex to avoid or minimize some of these consequences [1]. However, survivors who are not aware of the benefits of seeking help may become reluctant to seek help. Awareness of the timing for PEP may, therefore, create the agency for an individual to take adequate measures following exposure to sexual violence.

The findings further show that male respondents were more likely than their female counterparts to express favorable attitudes towards help-seeking for SGBV. However, previous studies show that men are more likely than women to express unwillingness or lack of motivation to seek help for stressful life events [42, 43]. The finding of this paper may be attributed to contextual factors such as women’s lack of autonomy and economic empowerment as well as low levels of education compared to men [25–27]. The proportion of women with no education was more than twice higher than that of men (73% and 34% respectively). It may also be possible that women are more likely to experience SGBV, therefore, have a more intimate understanding of how hard or uncomfortable it can be to disclose. The result suggests a more sensitized evaluation of people’s experiences with violence so as to provide appropriate support. There is also need to include non-literacy-based messaging in order to reach community members with no formal education.

Unlike women, the finding shows statistically insignificant association between attitude towards help-seeking for SGBV and most of the explanatory factors among men in the study area. The difference in the result between men and women may be attributed to a number reasons. Some of the men may have been ambivalence in their responses to the generic nature of the questions on attitude towards help-seeking for SGBV. For example, the statement, "it is important for rape survivors to report immediately to a health center and police", does not necessarily imply the respondent considers herself or himself as a survivor when responding. It may also be possible that some of the men may have considered female victims when answering questions on help-seeking given a high prevalence of SGBV among women. Research also shows that, sometimes, study participants may react differently to research questions on sensitive topics—such as SGBV, on the basis of their age, gender and previous or current personal experience amongst other personal traits [44].

The second part of this study examined the relationship between attitudes towards help-seeking and actual help-seeking behavior among those respondents who reported experiencing partner or non-partner violence in the last one month prior to interview date. Results indicated that the probability of seeking help for SGBV was higher among respondents with a favorable attitude towards seeking help for SGBV compared to those respondents with unfavorable attitude toward reporting. The findings are consistent with the hypothesis that a person’s attitudes toward seeking help determine actual help-seeking behavior [45]. Although this finding is indicative of a positive association between attitude towards and actual help-seeking behavior for SGBV, the results ought to be interpreted with caution given lack of prior information on respondent’s utilization of SGBV services.

### Limitations

The findings of the paper could be influenced by certain limitations. The study was based on cross-sectional data; it was therefore not possible to establish causality between independent and outcome variables. For instance, there could be a possibility of behavior fostering attitude, that is, past SGBV survivors who have benefitted from care from health facilities or the police help may be prompted to give positive responses. Respondents were asked about their perceptions of the importance and value of help-seeking at health facilities and police stations for SGBV in general without probing for personal beliefs and motivations underlying their attitudes. Such details can only be obtained through qualitative interviews. In addition, the study did not examine help-seeking attitude from informal settings, which may give a different result from the one presented here. Past studies reveal that informal sources of heal such as family members, and friends are the most preferred source of support [23].

In terms of measurement, the paper is based on quantitative scales that measure perceptions about SGBV norms in the community. However, a quantitative scale may not explain all or most of the variations in such attitude-related attributes. In particular, it was not possible to develop a robust measure that is appropriate for the humanitarian setting. Furthermore, including only heads of households may underestimate the prevalence of SGBV. Another limitation is that attitudes towards help-seeking for SGBV may be influenced by other factors not captured in this study such as the severity of violence [46], availability of services, and inequality in gender norms [23].

## Conclusion

SGBV remains a silent epidemic with many survivors in humanitarian settings concealing their experiences of abuse. The findings of the paper suggest that targeted interventions aimed at promoting awareness and progressive attitudes towards SGBV are likely to encourage positive help-seeking attitudes and behaviors in such contexts.

## Abbreviations

IPV: Intimate partner violence
ODK: Open Data Kit
PEP: Post exposure prophylaxis
SGBV: Sexual and gender-based violence
